# Bu Shen Huo Xue decoction promotes functional recovery in spinal cord injury mice by improving the microenvironment to promote axonal regeneration

**DOI:** 10.1186/s13020-022-00639-y

**Published:** 2022-07-12

**Authors:** Yonghui Hou, Dan Luo, Yu Hou, Jiyao Luan, Jiheng Zhan, Zepeng Chen, Shunmei E, Liangliang Xu, Dingkun Lin

**Affiliations:** 1 Department of Orthopedic Surgery, Guangdong Provincial Hospital of Chinese Medicine, The Second Affiliated Hospital of Guangzhou University of Chinese Medicine, No. 111 Dade Road, Guangzhou, 510120 Guangdong People’s Republic of China; 2 Guangzhou University of Chinese Medicine, No. 12, Jichang Road, Baiyun District, Guangzhou, 510405 Guangdong People’s Republic of China; 3 Lingnan Medical Research Center of Guangzhou University of Chinese Medicine, Guangzhou, 510405 Guangdong People’s Republic of China; 4 Department of Laboratory Medicine, Guangdong Provincial Hospital of Chinese Medicine, The Second Affiliated Hospital of Guangzhou University of Chinese Medicine, Guangzhou Higher Education Mega Center, 55 Neihuan Xi Road, Panyu District, Guangzhou, 510120 Guangdong People’s Republic of China; 5 Key Laboratory of Orthopaedics & Traumatology, The First Affiliated Hospital of Guangzhou University of Chinese Medicine, Guangzhou University of Chinese Medicine, Guangzhou, People’s Republic of China

**Keywords:** Spinal cord injury, Bu-Shen-Huo-Xue decoction, Microenvironment, Axonal regeneration, Inflammatory response

## Abstract

**Background:**

Bu-Shen-Huo-Xue (BSHX) decoction has been used in the postoperative rehabilitation of patients with spinal cord injury in China. In the present study, we aim to reveal the bioactive compounds in BSHX decoction and comprehensively explore the effects of BSHX decoction and the underlying mechanism in spinal cord injury recovery.

**Methods:**

The main chemical constituents in BSHX decoction were determined by UPLC–MS/MS. SCI mice were induced by a pneumatic impact device at T9–T10 level of the vertebra, and treated with BSHX decoction. Basso–Beattie–Bresnahan (BBB) score, footprint analysis, hematoxylin–eosin (H&E) staining, Nissl staining and a series of immunofluorescence staining were performed to investigate the functional recovery, glial scar formation and axon regeneration after BSHX treatment. Immunofluorescent staining of bromodeoxyuridine (BrdU), neuronal nuclei (NeuN) and glial fibrillary acidic protein (GFAP) was performed to evaluate the effect of BSHX decoction on neural stem cells (NSCs) proliferation and differentiation*.*

**Results:**

We found that the main compounds in BSHX decoction were Gallic acid, 3,4-Dihydroxybenzaldehyde, (+)-Catechin, Paeoniflorin, Rosmarinic acid, and Diosmetin. BSHX decoction improved the pathological findings in SCI mice through invigorating blood circulation and cleaning blood stasis in the lesion site. In addition, it reduced tissue damage and neuron loss by inhibiting astrocytes activation, and promoting the polarization of microglia towards M2 phenotype. The functional recovery test revealed that BSHX treatment improved the motor function recovery post SCI.

**Conclusions:**

Our study provided evidence that BSHX treatment could improve the microenvironment of the injured spinal cord to promote axonal regeneration and functional recovery in SCI mice.

## Introduction

Spinal cord injury (SCI) is a severe neurological disease mainly caused by physical trauma to the spinal cord [1, 2]. According to the pathophysiology progress, spinal cord injury can be divided into primary injury and secondary injury [3]. At the primary stage, physical trauma to the spinal cord causes blood-spinal cord barrier disruption, leading to hemorrhage and edema [4]. Ischemia and refusion result in neuronal and glial necrosis and apoptosis, axon degeneration and demyelination in the lesion epicenter, and initiate the secondary injury including inflammatory response [5]. Excessive inflammatory response and extravasation of infiltrating leukocytes cause the expansion of apoptosis from the lesion epicenter to the adjacent area, which aggravates tissue damage [6]. Microglia-mediated neuroinflammatory response at the lesion site may amplify the secondary injury after SCI [7]. Microglia have two different phenotypes, the classical M1 microglia which secrets pro-inflammatory cytokines and the alternative M2 microglia which secrets anti-inflammatory cytokines [8]. Therefore, converting classical M1 to M2-type microglia to diminish inflammatory response at the early stage can inhibit the tissue damage aggravation.

Then, the astrocytes, oligodendrocyte progenitor cells and endogenous neural stem cells (NSCs) in the penumbra of the lesion site become activated, and begin to proliferate, migrate to the lesion site to form the scar [9, 10]. And the fibrotic component of the scar contributes to the axon regeneration failure in the lesion core after SCI [11]. Neuron necrosis and apoptosis, as well as axon demyelination and loss in the lesion site lead to permanent neurological impairment. Propelling axon regeneration and regrowth across the lesion site helps to foster functional recovery after SCI [12]. However, the treatments for SCI are very limited until now. Therefore, developing new strategies and drugs is urgently needed to prevent secondary injury expansion and promote axon regeneration in the lesion core.

Traditional Chinese medicine theory considers that physical trauma to the spinal cord leads to damage to blood vessels and veins, and blood extravasation in the lesion site of the spinal cord, which leads to dysfunction between meridians and results in a series of clinical symptoms [13]. Blood stasis blocks hematopoiesis, which will further damage the governor vessel and lead to more disorder between the governor vessels and other meridians. Therefore, promoting blood circulation and removing blood stasis in the lesion site of the spinal cord will provide a beneficial microcirculation for spinal cord injury recovery. In addition, the kidney has been considered as the source of spinal cord according to the traditional Chinese medicine theory. Tonifying the kidney can benefit the essence and give birth to the marrow. If the essence in the kidney is sufficient, the marrow gets to raise [13]. Therefore, tonifying the kidney plays beneficial functions in spinal cord injury recovery. Bu-Shen-Huo-Xue (BSHX) decoction originates from ancient Chinese literature “Shangke Dacheng”. It has the effects of tonifying kidney and filling essence, removing stasis and clearing collaterals [14]. And BSHX decoction has been used in postoperative rehabilitation of patients with spinal cord injury in China [15].

In this study, we aimed to comprehensively explore the neuroprotective effects of BSHX decoction on spinal cord injury recovery as well as the underlying mechanism. The SCI mice were induced by a pneumatic impact device at T9–T10 level of the vertebra and administrated with BSHX decoction to evaluate its therapeutic effect. Then we performed immunofluorescent staining to analyze astrocytes activation, NSCs proliferation and differentiation to explore the related mechanism.

## Materials and methods

### BSHX decoction preparation

BSHX decoction is composed of nine Chinese herbs listed in Table 1. All herbs were purchased from Guangdong Provincial Hospital of Chinese Medicine. BSHX decoction can be divided into two parts, the Bu-Shen group used to tonify the kidney including Morindae Officinalls Radix, Curculiginis Rhizoma, Astragali Radix and Aconiti Lateralis Radix Praeparata, and the Huo-Xue group used to invigorate the circulation of blood including Salviae Miltiorrhizae Radix Et Rhizoma, Spatholobi Caulis, Paeoniae Radix Rubra, Angelicae Sinensis Radix and Chuanxiong Rhizoma. All herbs of BSHX decoction were mixed and soaked in 5 volumes of water (volume/weight) for 1 h, and then decocted and boiled for 30 min. The decoction was filtered and concentrated to 78 mL.

**Table 1:** Chinese herbs of BSHX decoction

Chinese name	English name	Latin name	Place of origin	Quantities (g)
Chi Shao	Paeoniae Radix Rubra	*Paeonia lactiflora Pall*	Neimenggu, China	15
Ba Ji Tian	Morindae Officinalis Radix	*Morinda officinalis How*	Guangdong, China	15
Dang Gui	Angelicae Sinensis Radix	*Angelica sinensis (Oliv.) Diels*	Gansu, China	15
Dan Shen	Salviae Miltiorrhizae Radix Et Rhizoma	*Salvia miltiorrhiza Bge*	Sichuan, China	15
Huang Qi	Astragali Radix	*Astragalus membranaceus (Fisch.)*	Gansu, China	60
Ji Xue Teng	Spatholobi Caulis	*Spatholobus suberectus Dunn*	Anhui, China	15
Chuan Xiong	Chuanxiong Rhizoma	*Ligusticum chuanxiong Hort*	Sichuan, China	15
Xian Mao	Curculiginis Rhizoma	*Curculigo orchioides Gaertn*	Guangdong, China	15
Shu Fu Zi	Aconiti Lateralis Radix Praeparata	*Aconitum carmichaelii Debx*	Sichuan, China	15

### Animals

C57BL/6 mice in 8 weeks were provided by Guangdong Medical Experimental Animal Centre. The mice were housed under a 12 h/12 h light/dark cycle at a controlled temperature and supplied with free food and water. All animal procedures were approved by the Ethics Committee of Guangzhou University of Chinese Medicine and performed according to the guidelines of the Chinese National Institute of Health (Guangzhou, China; Certificate No. 44005800013161).

### The main chemical constituents in BSHX decoction determined by UPLC–MS/MS

Identification analysis was performed on an Agilent 1100 UPLC system (Agilent Technologies Inc., United States) coupled with a triple quadrupole mass spectrometer system API4000. Samples were dissolved in methanol (HPLC grade), filtered with 0.22-μm filtration membrane, and separated on an Agilent poroshell 120 EC-C18 column (3 mm × 50 mm, 2.7 μm). The mobile phase system was water contained with 0.5% formic acid (A) and acetonitrile (C). The gradient elution procedure was as follows, 0–1 min, 5% C; 1–8 min, 5–25% C; 8–12 min, 25–60% C; 12–16 min, 60–100% C. The flow velocity was 0.6 mL/min, and the column temperature was 35 °C. The injection volume was 10 μL.

The mass spectrometry was set as follows: Spray voltage, 4.5–5.5 kv; the desolventize temperature, 450 °C; the desolventize gas, nitrogen; and desolventize gas (N2) flow, 1000 L/h.

**Fig. 1:**
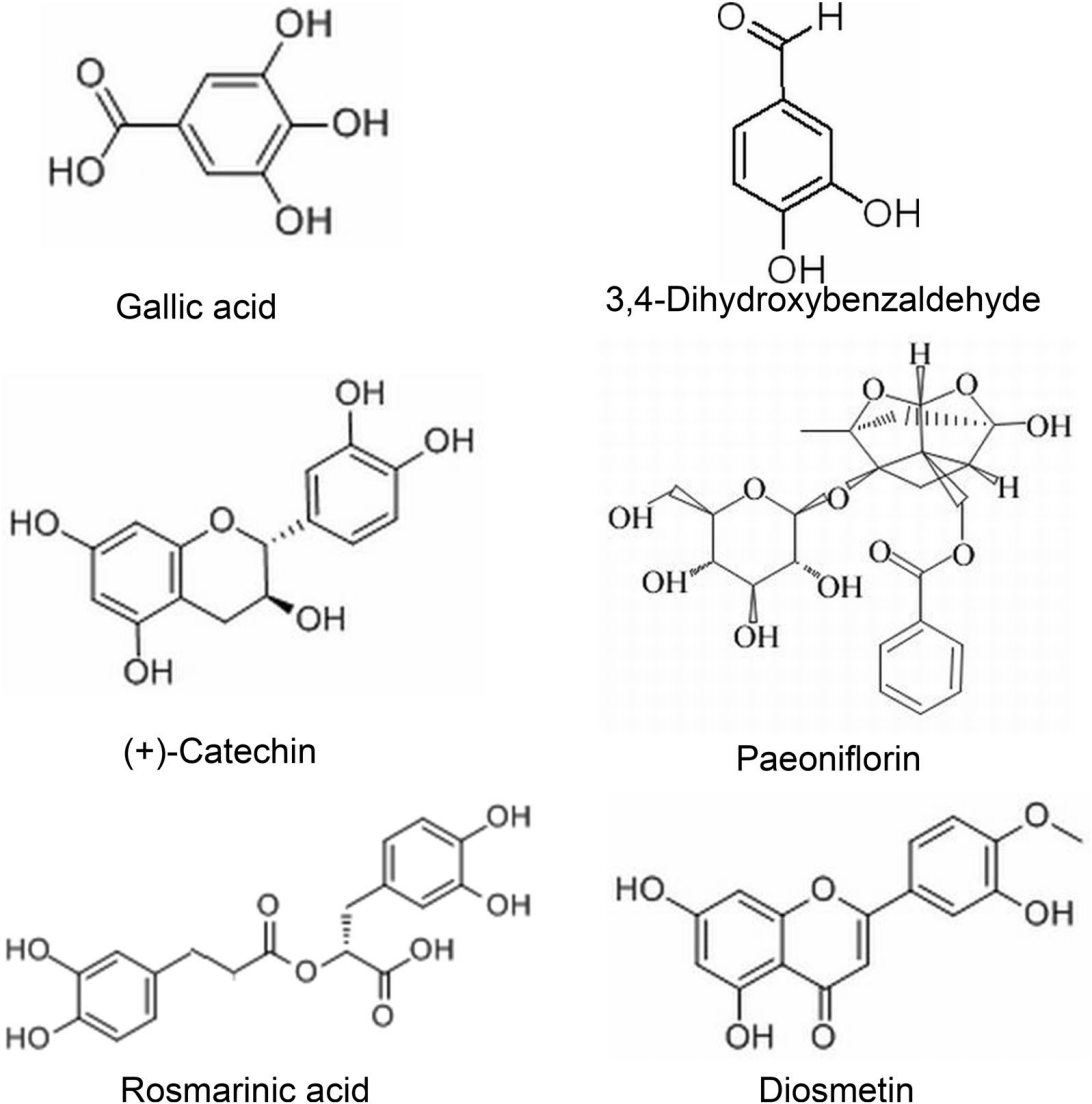
The structure of six main identified compounds in BSHX decoction

**Fig. 2:**
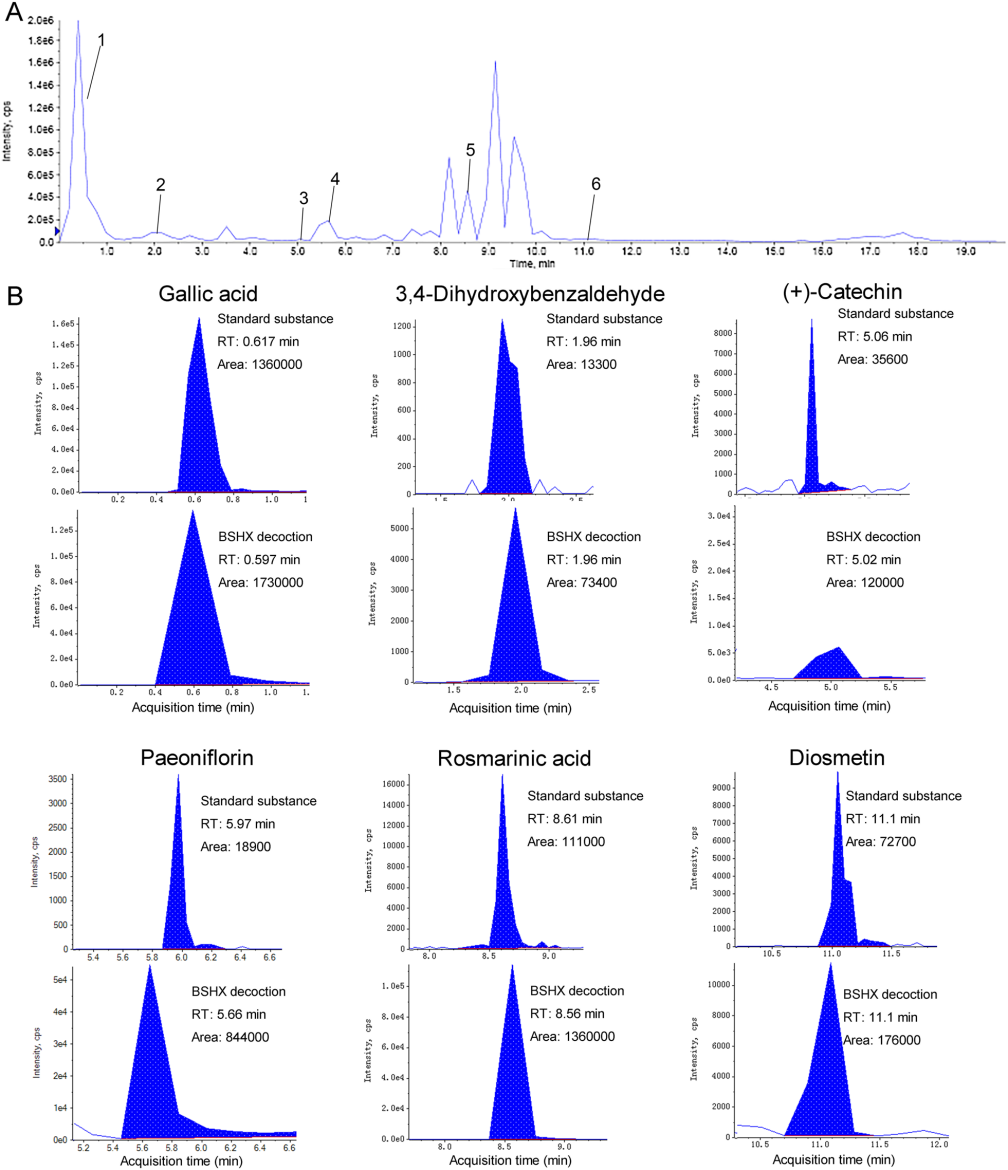
Base peak ion chromatogram of the six main compounds from BSHX decoction in negative ion mode by UPLC–MS/MS. **A** Total ion chromatogram from BSHX decoction in negative ion mode by UPLC–MS/MS. **B** Peak areas information of six compounds determined using the standard substance

The main compounds identified were Gallic acid, 3,4-Dihydroxybenzaldehyde, (+)-Catechin, Paeoniflorin, Rosmarinic acid, and Diosmetin, which were shown in Figs. 1 and 2, as well as Table 2.

**Table 2:** Data for the main compounds identified by ULPC–MS/MS

No	Identification	Retention time (min)	Precursor ion (*m/z*)	Molecular formula
1	Gallic acid	0.597	168.96	C_7_H_6_O_5_
2	3,4-Dihydroxybenzaldehyde	1.96	136.8966	C_7_H_6_O_3_
3	(+)-Catechin	5.02	288.9681	C_15_H_14_O_6_
4	Paeoniflorin	5.66	479.1	C_23_H_28_O_11_
5	Rosmarinic acid	8.56	358.9589	C_18_H_16_O_8_
6	Diosmetin	11.1	298.9089	C_16_H_12_O_6_

### SCI model and treatment

The SCI mice model was induced under sterile condition according to Allen’s methods as previously described [16]. After anesthetized with 1% pentobarbital sodium (50 mg/kg) by intraperitoneal injection, laminectomy was performed to expose the spinal cord at T9–T10 level of the vertebra. The SCI model was induced by a pneumatic impact device as our previous study described [16]. After surgery, manually bladder expression was performed twice a day until the reflex bladder emptying of mice recovered. A total of 60 males C57BL/6 mice were randomly divided into sham, SCI and BSHX groups. The mice in the sham group underwent laminectomy without SCI. According to clinical dose and based on the calculation conversion ratio of human to mouse [17], a dose of 200 μL BSHX decoction was given to mice daily. Mice in BSHX groups received 200 μL preheated BSHX decoction by oral route once a day post-injury. The sham and SCI groups received an equal volume of saline orally once a day.

### Functional behavior evaluation

Hindlimb locomotor function was assessed in an open field using Basso–Beattie–Bresnahan (BBB) locomotion scale [18]. The BBB tests, ranging from 0 to 21 (0, complete hindlimb paralysis; 21, normal locomotion) based on hindlimb joint movement and coordination, were conducted 1, 3, 7, and 14 days after SCI.

### Paraffin sections preparation

For immunofluorescent staining and histological assessment, the injured spinal cord samples were dissected out after transcardial perfusion with normal saline and 4% paraformaldehyde, and fixed in 4% paraformaldehyde (PFA) for 2 days at room temperature. And then dehydrated sequentially with 70%, 80%, 95% and 100% ethanol. Spinal cord samples were embedded in paraffin with appropriate orientation. The paraffin-embedded spinal cord was sectioned at 5 μm using a microtome (Leica, Germany) for hematoxylin–eosin (H&E) staining, Nissl staining and immunofluorescence staining.

### Immunofluorescence staining

The sections were dewaxed in xylene three times each for 5 min and rehydrated through a series of ethanol of decreasing concentrations (100% to 95% to 80% to 70% ethanol and then tap water once, each step for 5 min). After the sections were heated by microwave to near boiling for 20 min in antigen retrieval buffer (10 mM citric acid, pH 6.0), all sections were cooled down to room temperature, and blocked in PBS with 10% normal horse serum at room temperature for 1 h, and then incubated with the following primary antibody at 4 °C overnight: glial fibrillary acidic protein (GFAP, 1:500; Boster Biological Engineering Co.), Nestin (1:200; Abcam) microtubule-associated protein 2 (MAP2, 1:200; Boster Biological Engineering Co.), growth-associated protein 43 (GAP43, 1:200; NOVUS), myelin basic protein (MBP, 1:200; NOVUS), ionized calcium binding adaptor molecule 1 (Iba-1, 1:200; Cell Signaling Technology), CD163 (1:30; Santa Cruz) and CD68 (1:300; Boster Biological Engineering Co.). The secondary antibodies tagged with different Alexa fluor^®^ fluorochrome diluted in PBS with 10% normal horse serum were used to detect the corresponding primary antibody. The immunostained sections were examined under a fluorescence microscope (Olympus IX73).

### Primary culture of NSCs from spinal cord and treatment

NSCs were extracted from embryos of pregnant C57BL/6 mice at embryonic day 12.5 (E12.5) according to the protocol [19]. Briefly, spinal cords were isolated from mouse embryos and cut into small pieces, and then digested in 1 mL HBSS with 1 mg/mL hyaluronidase (H3884; Sigma), 2 mg/mL trypsin (T4799; Sigma), and 300 µg/mL DNase I (D4527; Sigma) at 37 °C for 30 min. Then added 6 mL of HBSS to stop the enzymatic reaction, and centrifuged at 380*g* for 5 min. The cell pellet was resuspended in 0.6 mL of HBSS with 1.5 mg/mL trypsin inhibitor (T9003; Sigma), and filtered through a 40-µm cell strainer. The cell suspension was spun down at 380 g for 5 min. At last, the cell pellet was re-suspended in DMEM/F12 medium with 10 ng/mL FGF2 (Peprotech), 10 ng/mL EGF (Peprotech) and 2 µg/mL Heparin and seeded into Petri dishes. Half of the culture medium was refreshed every 2 days afterward. The neurospheres were subcultured into single cells after 5 to 6 days. The morphology of spheres was characterized under a phase-contrast microscope and immunostained with Nestin, NeuN and GFAP.

To initiate neuronal differentiation, the cells were seeded into a 24-well plate precoated with poly-d-lysine (P7886; Sigma), and incubated with neuronal induction media composed of DMEM/F12, 2% B27 (12587010; Gibco), 1% GlutMAX (35050061; Gibco), and 1% FBS (10100139C; Gibco) with BSHX (1, 10, and 100 μg/mL) or without BSHX for 7 days. Half of the medium with indicated BSHX or without BSHX was changed every 3 days. And then cells were fixed and immunostained with GFAP and NeuN.

### Bromodeoxyuridine labeling

For bromodeoxyuridine (BrdU) labeling, BrdU (B5002, Sigma; 1 μmol/L/g body weight) was injected intraperitoneally into mice 2 h before sacrifice or added into the culture medium (10 μM) 1 h before fixation. Sections or cells were pre-incubated with 2 N HCl at 37 °C for 1 h to expose the BrdU within proliferating cells before incubating with primary antibodies. After BrdU labeling, the numbers of proliferating cells (BrdU positive cells) and the total number of cells (DAPI positive cells) on each section or cell culture were counted.

### Statistical analysis

Statistical analysis was performed using GraphPad Prism 8 software (GraphPad Software Inc.). All statistic results were presented as the means ± SD. One-way ANOVA was used to compare more than two groups, and an unpaired Student’s t-test was used to compare two groups. For statistical analysis, *P* < 0.05 was considered statistically significant (expressed as *P < 0.05 or **P < 0.01).

## Results

### BSHX decoction reduced tissue damage and promoted functional recovery after SCI

**Fig. 3:**
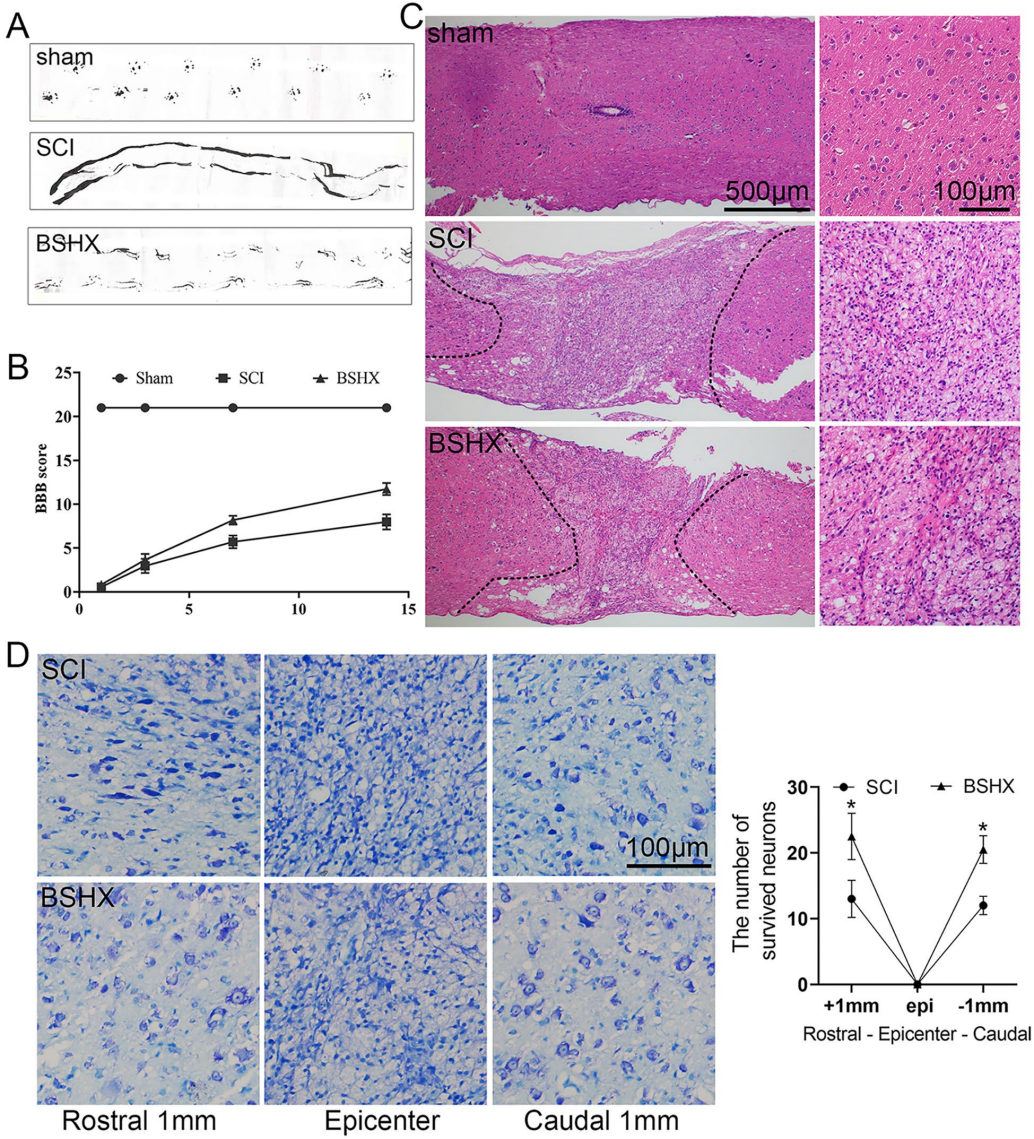
BSHX decoction improved pathological and functional recovery after SCI. **A** Footprint analysis for the different groups at day 14 after SCI. **B** BBB scores at 1, 3, 7 and 14 days after SCI. **C** Representative images showing H&E staining in longitudinal sections at day 14 after SCI. **D** Nissl staining showed the survival neurons in different groups at different distances from the epicenter of the lesion site. All experiments were performed in triplicated and data were presented as means ± SD, n = 3 per group. *P < 0.05

To examine the effect of BSHX decoction on motor function recovery after SCI, footprint analysis and BBB rating scale were performed for 14 days. Footprint analysis showed that SCI mice displayed incongruous gait and extensive dragging of both hindlimbs. Whereas in the BSHX decoction treated group, the mice exhibited relatively congruous gait at day 14 after SCI (Fig. 3A). And the BBB scores decreased in SCI and BSHX groups at 1, 3, 7, and 14 days compared to those in the sham group. At day 14, BSHX treated mice exhibited higher BBB scores than those in the SCI group (Fig. 3B). To assess the extent of tissue preservation at 14 days after SCI, we performed H&E staining and Nissl staining using spinal cord sections. Compared with the sham group, severe damage with malformation and cavity were observed in the lesion site and penumbra of the spinal cord with SCI. BSHX decoction treatment decreased the lesion area (Fig. 3C). Nissl staining for the survival neurons showed that motor neurons were lost in the epicenter of the lesion site and more neurons were observed in the penumbra region of the lesion site after BSHX treatment (Fig. 3D). These data indicated that BSHX treatment improved motor function by reducing tissue damage after SCI.

### BSHX treatment promoted cell proliferation at the early stage of SCI

**Fig. 4:**
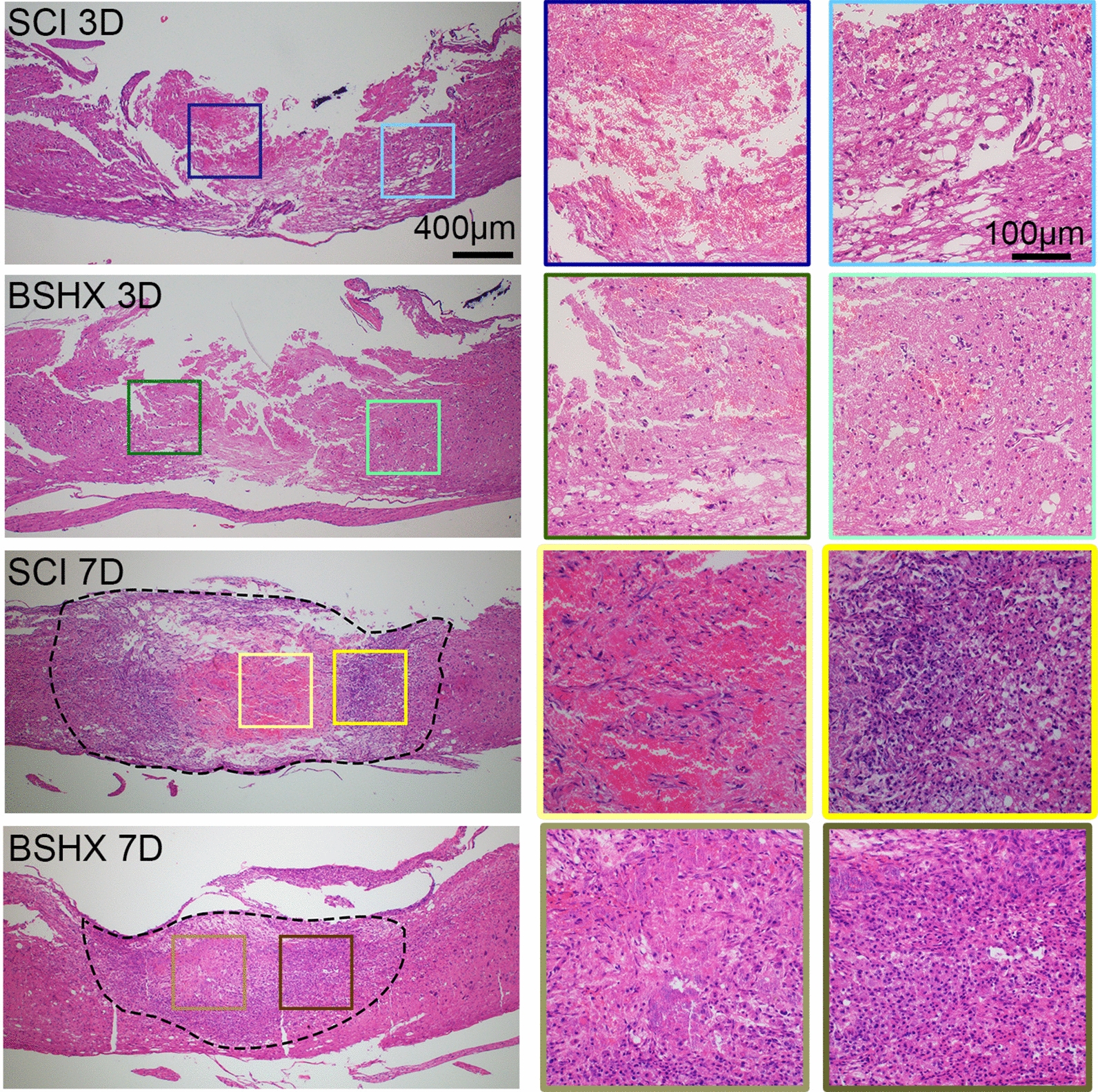
BSHX decoction promoted histological morphology improvement in the early stage of SCI. Representative image from H&E staining showed the histological morphology improvement in the injured spinal cord at day 3 or day 7 after SCI

**Fig. 5:**
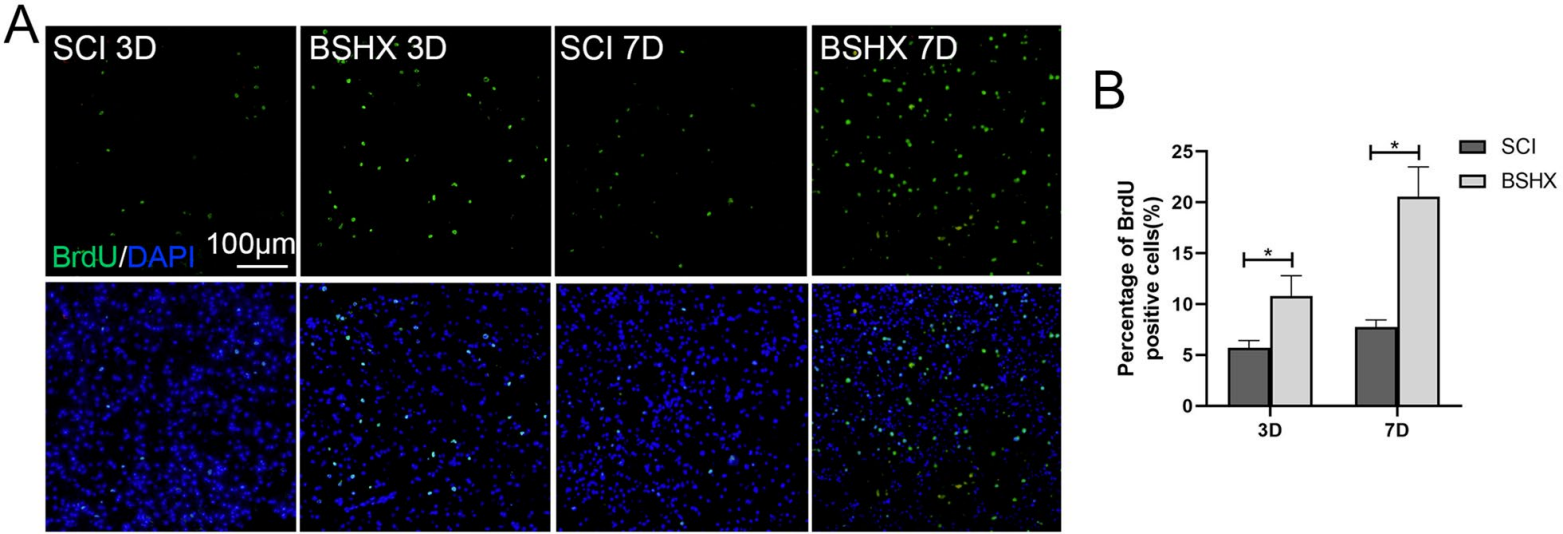
BSHX decoction promoted cell proliferation in the early stage of SCI. **A** BrdU labeling was performed to analyze the cell proliferation. **B** Quantification of BrdU positive cells. All experiments were performed in triplicated and data were presented as means ± SD, n = 3 per group. *P < 0.05, **P < 0.01

According to the Traditional Chinese medicine theory, BSHX decoction promotes blood circulation to remove blood stasis in the lesion site. To examine the effect of BSHX decoction on blood stasis at the early stage of SCI, H&E staining (Fig. 4) and BrdU labeling (Fig. 5) were performed to evaluate the blood stasis and cell proliferation in the early stage of SCI. It has been reported that physical trauma to the spinal cord causes glial and neuronal necrosis in the lesion epicenter, and axons shearing, degeneration along with the lesion [20]. As shown in Fig. 4, cellular debris and red blood cells occupied the lesion epicenter at day 3 in SCI and BSHX groups after SCI. And in the penumbra of the lesion site, obvious malformation and cavity were observed in the SCI group; while the cavitation caused by bleeding decreased and tissue was relatively intact after BSHX treatment. At day 7, the epicenter of the lesion in the SCI group was replete with red blood cells. Whereas in the BSHX group, much more cells but not red blood cells were observed at the lesion site. It has been reported that oligodendrocytes progenitor cells, astrocytes, and NSCs become activated, and begin to proliferate and migrate to the lesion margin after SCI [21]. Compared with the SCI group, the number of BrdU positive cells in the lesion penumbra was increased at day 3 and 7 in the BSHX groups (Fig. 5). These data indicated that BSHX treatment reduced the number of red blood cells in the lesion site and enhanced cell proliferation to prevent the expansion of injury and improve the pathology.

### BSHX decoction reduced astrocyte reactivity after SCI

**Fig. 6:**
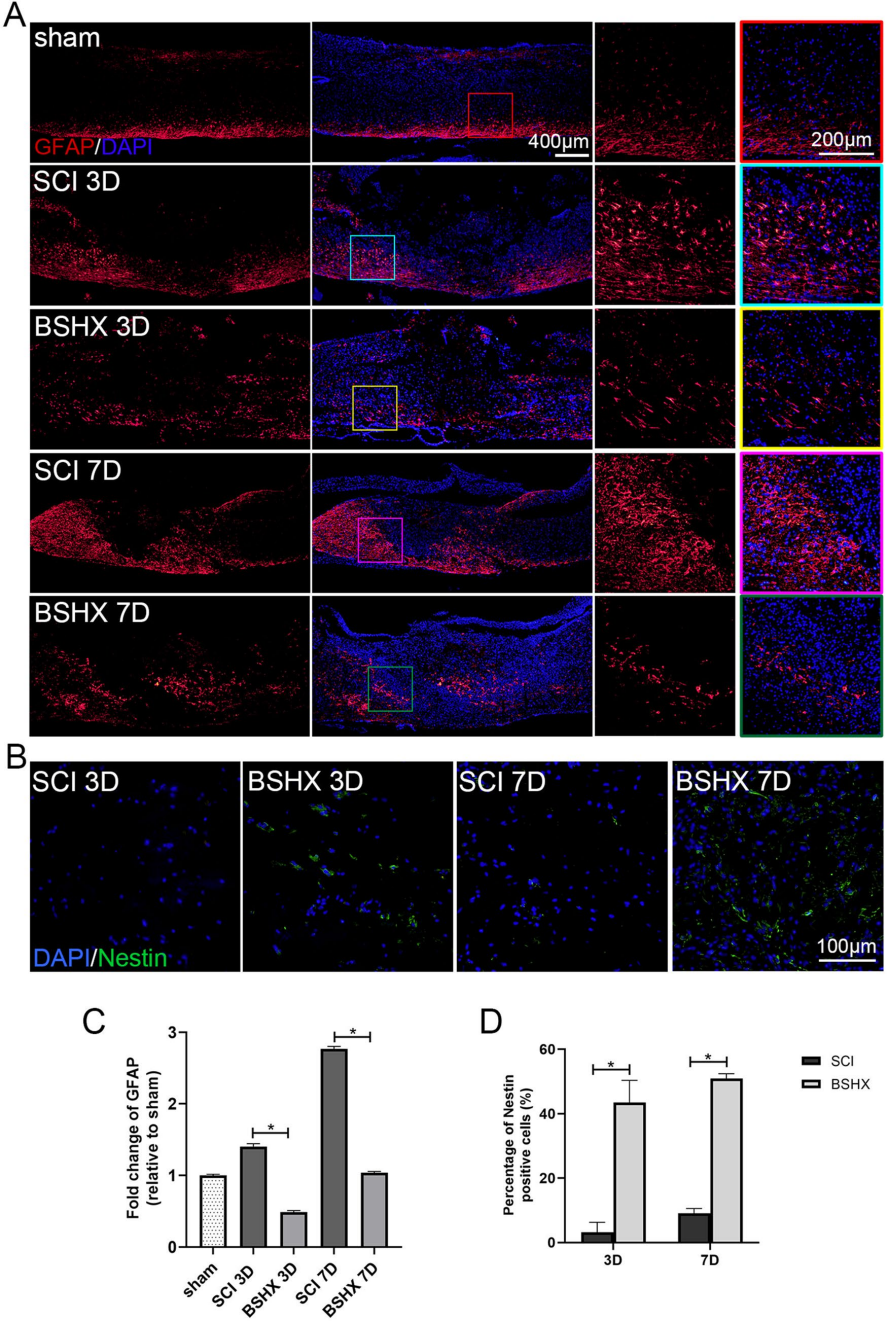
BSHX decoction reduced reactive astrocytosis after SCI. **A** Immunofluorescence images showed the reactive astrocytosis (GFAP, red) at day 3 and 7 after SCI. **B** Immunofluorescence images showed the neural stem cell (Nestin, green) in the lesion epicenter at day 3 and 7 after SCI. **C** Quantitative polymerase chain reaction (qPCR) showing the expression of GFAP after SCI. **D** Quantification of Nestin positive cells in the lesion epicenter. All experiments were performed in triplicated and data were presented as means ± SD, n = 3 per group. *P < 0.05, **P < 0.01

Following SCI, the reactive astrocytosis allowed the densely bundled barrier to form in the lesion site at day 14 after SCI [22], which was referred to as the glial component of the scar. On one hand, the reactive scar-forming astrocytes help to resolve the inflammation; on the other hand, they contribute to the chronic inhibitory structure to axon regeneration [23]. To elaborate on the effect of BSXH decoction on astrocyte reactivity, immunostaining of GFAP was performed to evaluate the astrocyte reactivity in and near the lesion site at day 3 and 7 after SCI (Fig. 6A). Compared with sham group, GFAP-positive cells were increased and mainly distributed in the lesion penumbra at day 3 and 7 in the SCI group. Whereas after BSHX treatment, GFAP-positive cells reduced and dispersed around the lesion site. And quantitative polymerase chain reaction (qPCR) result showed BSHX treatment inhibited GFAP mRNA expression (Fig. 6C). It has been reported that ependymal cells display neural stem cells properties after SCI and generate more astrocytes, a small number of oligodendrocytes and a few neurons [24]. Immunostaining of Nestin was used to illustrate the neural stem cell distribution in the lesion epicenter at day 3 and 7 after SCI (Fig. 6B). After BSHX treatment, Nestin-positive cells were observed in the lesion epicenter at day 3, and cell number increased at day 7 (Fig. 6D). Therefore, BSHX treatment reduced astrocyte reactivity and promoted neural stem cells to migrate to the lesion epicenter.

### BSHX decoction reduced fibrotic scar tissue and promoted axon regeneration after SCI

**Fig. 7:**
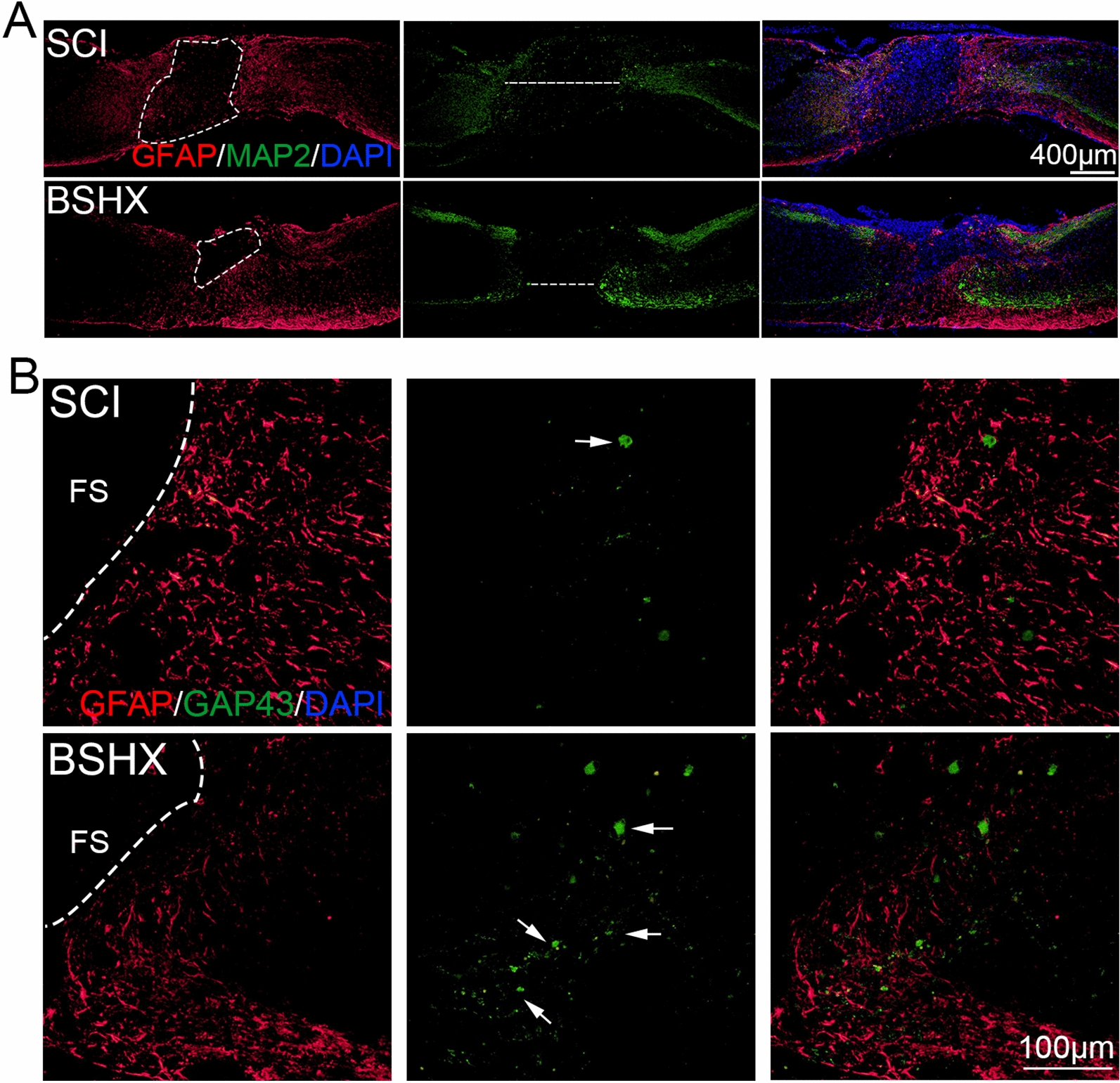
BSHX decoction decreased the damage of tissue and promoted axon regeneration after SCI. **A** Co-immunofluorescence images showed GFAP (red) and MAP2 (green) at day 14 after SCI. **B** Co-immunofluorescence images showed the axonal regeneration (GFAP, red; GAP43, green) in the lesion site at day after SCI. *FS* Fibrotic scar

Given that glia scar formation is a critical progress of SCI pathology and vital for functional recovery after SCI [25], we performed immunostaining of GFAP and MAP2 to evaluate the glial scar and neuron loss in the injured spinal cord at day 14 after injury. In the SCI group, a dense fibrotic scar, surrounded by activated astrocytes formed in the lesion area at day 14. And neuron loss located at the glial scar was observed in the injured spinal cord. Compared with the SCI group, the fibrotic scar area and neuron loss region decreased in the injured spinal cord after BSHX treatment (Fig. 7A). GAP43, which is expressed at high levels in neuronal growth cones during axonal regeneration [26], was used to evaluate the axonal regeneration in the injured spinal cord by immunostaining (Fig. 7B). In the SCI group, few GAP43-positive axons were observed in GFAP-positive glial scar, whereas more GAP43-positive axons were observed in the BSHX group. These data showed that BSHX treatment could reduce fibrotic scar area and promote axonal regeneration in the lesion site after SCI.

### BSHX decoction promoted macrophage polarization from M1 to M2

**Fig. 8:**
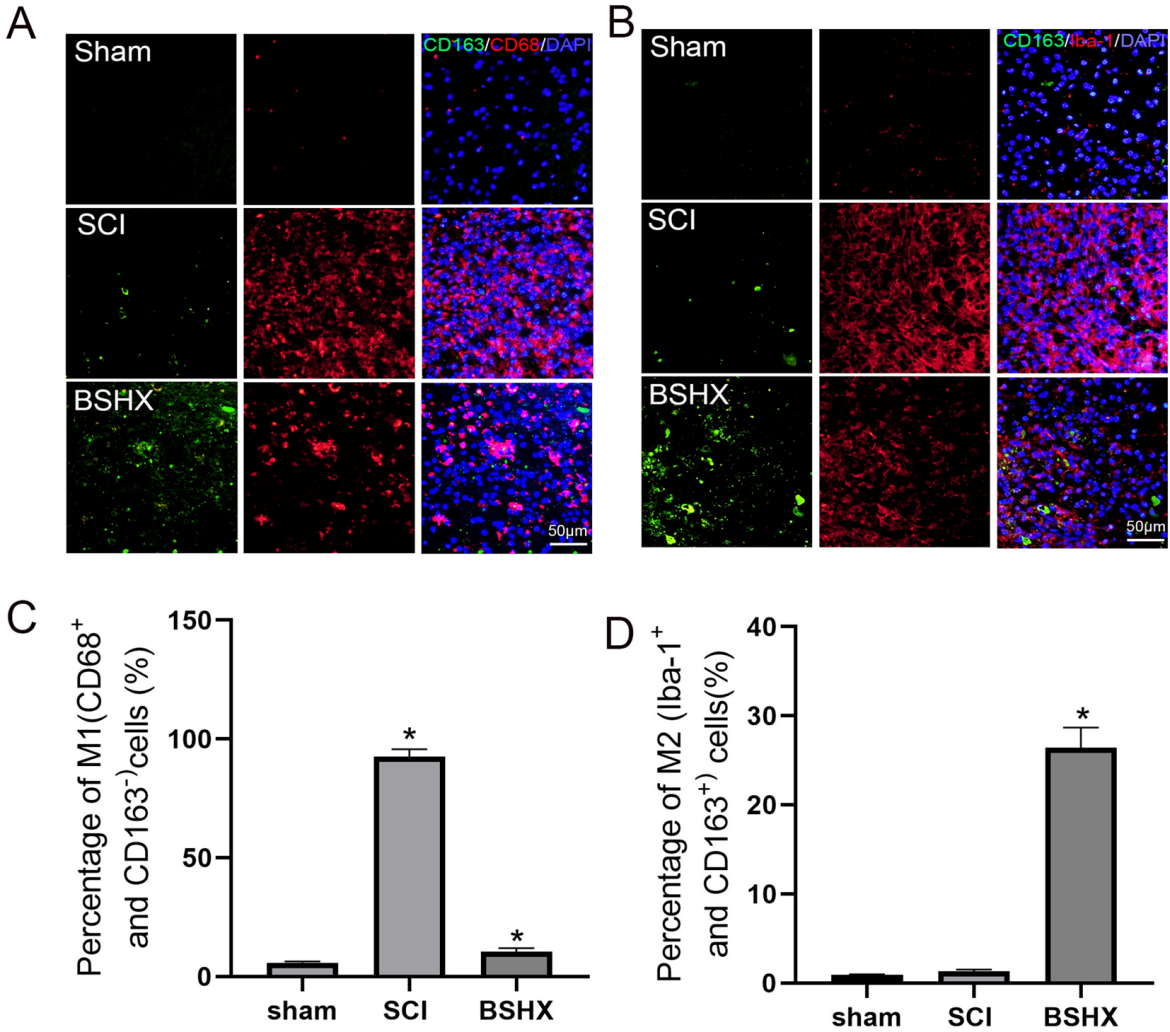
BSHX decoction promoted microglia polarization from M1 to M2. **A** Immunofluorescent staining of CD68 (red)/CD163 (green) in the lesion site of the spinal cord 14 days after SCI. **B** Immunofluorescent staining of Iba-1 (red)/CD163 (green) in the lesion site of the spinal cord 14 days after SCI. **C**, **D** Quantification the number of M1 (CD68^+^/CD163^−^) or M2 (Iba-1^+^/CD163^+^) cells in spinal cord. All experiments were performed in triplicated and data were presented as means ± SD, n = 3 per group. *P < 0.05, **P < 0.01

Around 14 days after SCI, activated microglia and proliferating fibroblasts occupy the lesion core of the glial scar [27]. And the M1 type microglia is dominant and persist, which may induce axon damage [28]. Therefore, converting classical M1 to relative M2-type microglia may be a beneficial intervention to diminish inflammatory response and enhance axon regeneration in the lesion site. To elaborate the distribution of activated microglia, we performed Iba-1 (microglia marker) and CD163 (M2-associated marker), CD68 (microglia activation marker) and CD163 double immunostaining to identify M1 and M2 microglia. As shown in Fig. 8A and B, a few cells were Iba-1 positive or CD68 positive in the sham group. Whereas in the SCI group, most of the cells were Iba-1 positive and CD68 positive and a few cells were CD163 positive in the lesion site, indicating that microglia were activated and occupied the lesion core and most of them were classical M1 type not M2 (Fig. 8C). While after BSHX treatment, the number of Iba-1 positive and CD68 positive cells decreased and more cells were Iba-1 and CD163 positive, which indicated the number of M2 type microglia increased (Fig. 8D). These results indicated that BSHX treatment prevented microglial activation and converted activated microglia from classically reactive M1 to M2 phenotype in the injured spinal cord, which diminished excessive inflammatory response and provided a beneficial microenvironment for axon regeneration in the injured site.

### BSHX decoction promoted remyelination

**Fig. 9:**
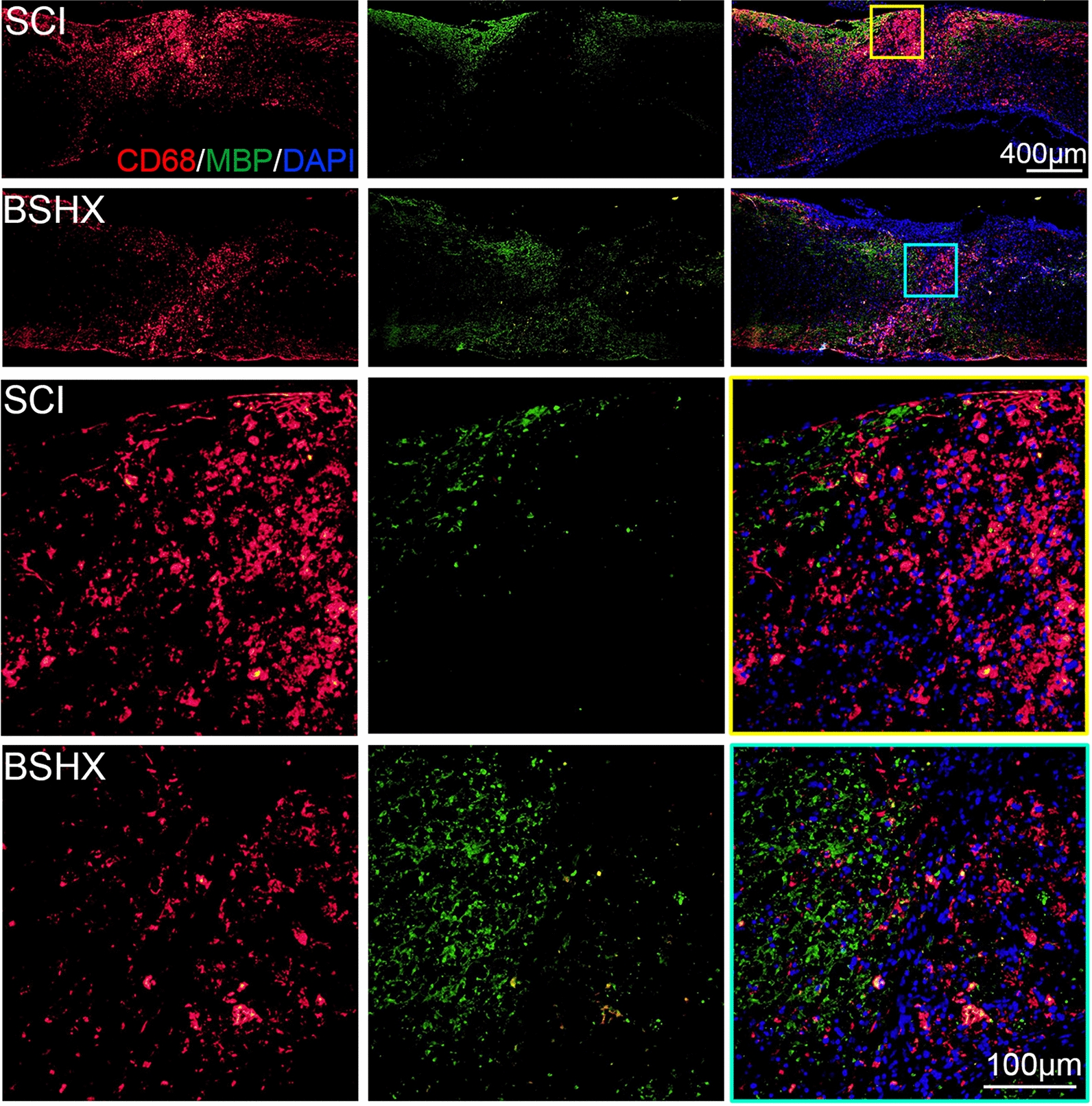
BSHX decoction promoted remyelination activity. Immunofluorescent staining of activated microglia (CD68 positive, red) and oligodendrocytes (MBP positive, green) in the lesion site of the spinal cord 14 days after SCI

Enhancing oligodendrocyte survival and remyelination are helpful for functional recovery after SCI [29]. We performed immunofluorescent staining of CD68 and MBP to evaluate the effect of BSHX treatment on remyelination after SCI (Fig. 9). In the SCI group, CD68 positive microglia occupied the glial scar and MBP positive oligodendrocytes distributed around the fibrotic scar 14 days after SCI, consisting with that around 14 days after SCI activated microglia occupy the lesion core of the glial scar and oligodendrocytes occupy the penumbra of the lesion site [3]. Whereas after BSHX treatment, CD68 positive microglia were reduced and MBP positive oligodendrocytes were increased in the lesion site.

### BSHX decoction promoted NSCs proliferation and differentiation into neurons

**Fig. 10:**
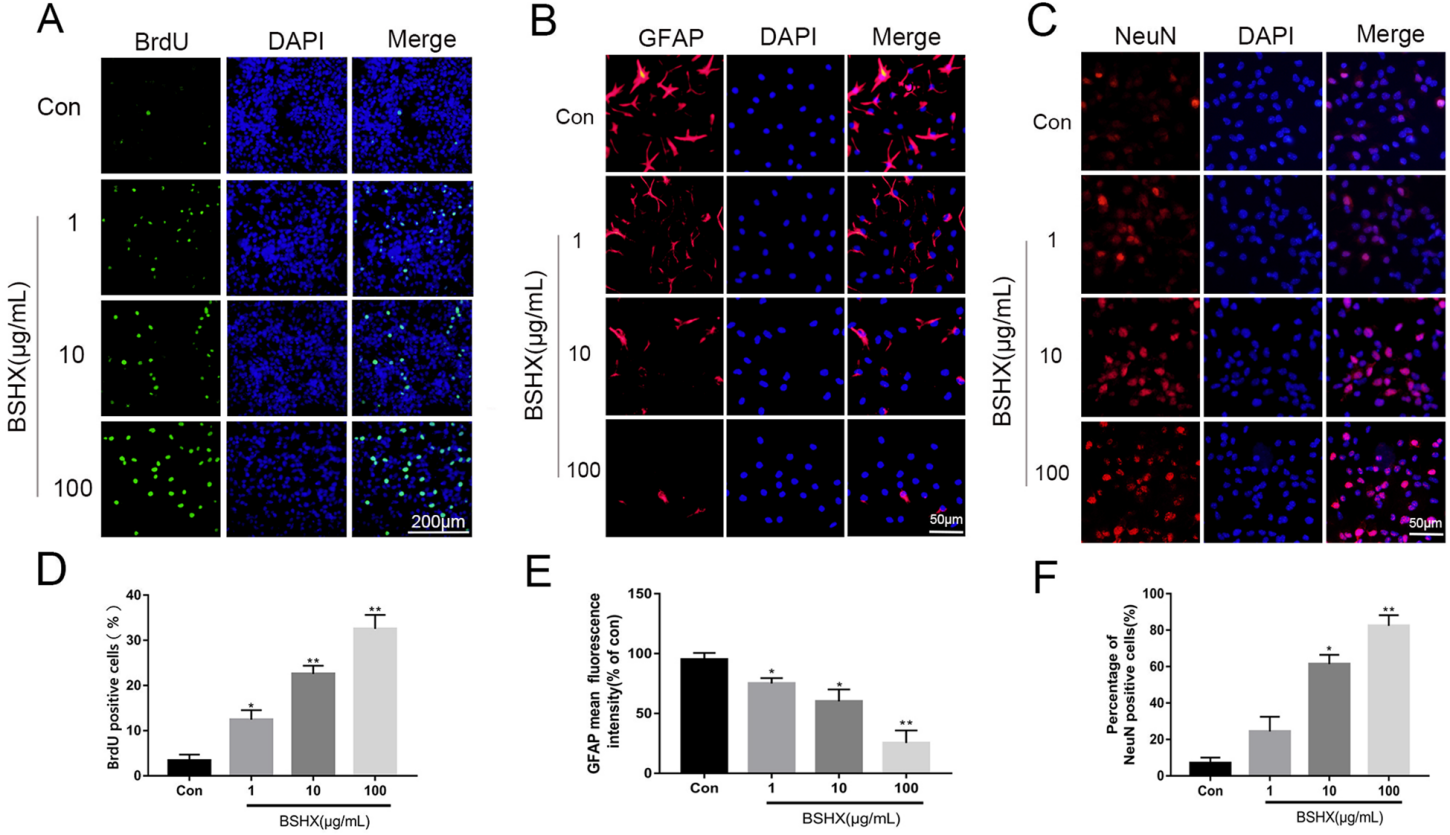
BSHX decoction promoted NSCs proliferation and neuronal differentiation. **A**, **D** BrdU labeling analysis in NSCs proliferation. **B**, **E** Immunofluorescent staining of GFAP after neural differentiation. **C**, **F** Immunofluorescent staining of NeuN after neural differentiation. All experiments were performed in triplicated and data were presented as means ± SD, n = 3 per group. *P < 0.05, **P < 0.01

In the uninjured spinal cord, ependymal cells referred to as endogenous NSCs remain quiescent [30]. After SCI, Endogenous NSCs rapidly proliferate, migrate to the injured site, and differentiate into astrocytes, oligodendrocytes, and neurons [31]. The loss of neurons in the injured site leads to dysfunction after SCI [32]. Therefore, promoting endogenous NSCs to differentiate into neurons may be an effective strategy for functional recovery. We performed immunofluorescence staining to evaluate the effect of BSHX on NSCs proliferation and differentiation (Fig. 10). As shown in BrdU labeling analysis, BrdU positive cells increased significantly with the indicated concentration after BSHX treatment. After 7 days of neural differentiation, NSCs mainly differentiate into astrocytes (GFAP positive) without BSHX treatment. While in BSHX group, NSCs mainly differentiate into NeuN positive neurons. These results indicated that BSHX treatment promoted NSCs proliferation and induced NSCs to differentiate into neurons, not astrocytes.

## Discussion

BSHX decoction has been reported to promote the rehabilitation of patients with spinal fracture and spinal cord injury. It is conducive to reducing pain, improving the motor function of patients after surgery and reducing the levels of postoperative inflammatory factors [15]. Our results also demonstrated that BSHX decoction improved SCI mice functional recovery through reducing lesion area and increasing the number of neurons with normal morphology and Nissl bodies.

To clarify the mechanism of BSHX decoction on spinal cord injury treatment, we performed UPLC–MS/MS to specify the main bioactive ingredients in BSHX decoction. The main compounds identified were Gallic acid, 3,4-Dihydroxybenzaldehyde, (+)-Catechin, Paeoniflorin, Rosmarinic acid, and Diosmetin. Numerous studies have reported that these bioactive ingredients showed a neuroprotective effect in neurological disease. Paeoniflorin attenuates neuroinflammation and inhibits apoptosis to ameliorate neuropathic pain [33], and it shows neuroprotection in spinal cord injury by reducing neuroinflammation induced by NF-κB [34]. Rosmarinic acid suppresses oxidative stress and inflammation through modulating the Nrf2/HO-1 and TLR4/NF-κB pathways to improve locomotor recovery in a rat model of SCI [35]. Although these bioactive ingredients show the neuroprotective effect in neurological disease, the exact mechanism of these bioactive ingredients working together in spinal cord injury treatment is still unclear.

Following SCI, hemorrhaging and edema in the lesion site cause ischemia and reperfusion injury in the cell dense area which result in glial and neuron apoptosis and the release of proinflammatory factors [36]. At this point, red blood cells, cellular debris, necrotic and apoptotic glia and neurons occupy the lesion epicenter. This triggers the secondary injury which leads to the expansion of tissue damage from the lesion site to the adjacent tissues [3]. Thus, promoting blood circulation to remove blood stasis at the early stage will be an effective intervention to alleviate secondary injury. In traditional Chinese medicine, tonifying the kidney and activating blood circulation has been considered as the basic method for the treatment of SCI. Here, we demonstrated that BSHX treatment cleared away the red blood cells and cellular debris in the lesion site, and reduced the cavitation caused by bleeding at the early stage of the injury. Besides, BrdU labeling result showed that BSHX treatment increased the rate of cell proliferation at the early stage of the injury. Thus, BSHX treatment preserved tissue integrity and promoted wound healing through tonifying the kidney and activating blood circulation.

Latent NSCs located in the ependymal layer remain quiescent in the intact spinal cord. These ependymal cells are activated and begin dividing rapidly after SCI [37]. And they give rise to large amounts of astrocytes that contribute to the glial scar formation, a small number of oligodendrocytes and a few neurons [38]. In addition, the loss of neurons in the lesion site is the main reason for the limited recovery after SCI [30]. Thus, promoting NSCs differentiation into functional neurons rather than astrocytes is an effective strategy to improve SCI functional recovery. The immunostaining of GFAP or Nestin showed that BSHX treatment prevented astrocytosis and suppressed astrocyte differentiation, and increased the number of NSCs in the lesion epicenter at the early stage of SCI. Given that astrocyte reactivity plays a critical role in glial scar formation, we performed immunostaining to evaluate the scar formation after SCI. The results showed that the fibrotic component area of the glia scar and neuron loss were reduced at day 14 post-SCI. Therefore, BSHX treatment provided a favorable microenvironment for axon regeneration through clearing red blood cells and cellular debris in the lesion site, preventing reactive astrocytosis, suppressing astrocytes differentiation, and reducing tissue damage.

Then, we further investigated the axon regeneration in the injured spinal cord after BSHX treatment. The immunofluorescent staining showed that more GAP43-positive axons were observed in the glial scar at day 14 post-SCI after BSHX treatment, indicating BSHX treatment promoted axon regrowth. In addition, inflammation leads to secondary injury and releases axon regeneration inhibitors to the microenvironment after SCI [7]. Thus, it is critical to find an intervention to inhibit inflammatory response after SCI. Microglia are macrophages which are present in the brain and spinal cord. Microglia participate in the inflammatory response and play an important role in neuroinflammation in central nervous system [39]. After SCI, microglia are rapidly activated within minutes and trigger the inflammatory response, which contributes to the expansion of tissue damage [40]. Classically activated M1 type microglia dominate and release proinflammatory cytokines whereas alternatively activated M2 showed a neuroprotective effect in spinal cord injury [40]. We found that BSHX treatment not only suppressed microglia activation but also converted activated microglia from M1 to M2 type and activated the remyelination within the injured spinal cord, indicating that BSHX can alleviate the inflammatory response and enhance remyelination after SCI.

According to the effects of BSHX treatment in SCI mice, we hypothesized that BSHX may contribute to NSCs proliferation and neural differentiation. And our results showed that BSHX treatment promoted NSCs proliferation and induced NSCs to differentiate into neurons rather than astrocytes in vitro, which was consistent with our finding that BSHX treatment inhibited astrocytes reactivity, reduced neuron loss and promoted axon regrowth in the lesion epicenter. However, we acknowledged that more detailed mechanisms underlying BSHX-mediated NSCs differentiation and axonal regeneration are still needed in further study.

## Conclusion

Taken together, this study provided evidence showing that BSHX treatment promoted functional recovery in SCI mice through improving the microenvironment to promote axonal regeneration. BSHX decoction improved the microenvironment through invigorating blood circulation to clean blood stasis in the lesion site, reduced tissue damage and neuron loss by suppressing astrocytes reactivation and inhibiting inflammatory response, and promoted axonal regeneration and remyelination. Our findings provide evidence that BSHX treatment is an effective intervention to improve functional recovery after SCI and can be used in the postoperative rehabilitation of patients with spinal cord injury.

## Data Availability

The data can be requested from the author upon reasonable request.

## Abbreviations

BBB: Basso–Beattie–Bresnahan
BrdU: Bromodeoxyuridine
BSHX: Bu-Shen-Huo-Xue
GAP43: Growth-associated protein 43
GFAP: Glial fibrillary acidic protein
H&E staining: Hematoxylin–eosin staining
Iba-1: Ionized calcium binding adaptor molecule 1
MAP2: Microtubule-associated protein 2
MBP: Myelin basic protein
NeuN: Neuronal nuclei
NSCs: Neural stem cells
PFA: Paraformaldehyde
SCI: Spinal cord injury
Tuj1: Neuron-specific class III beta-tubulin
